# Bioenergetics of acquired cisplatin resistant H1299 non-small cell lung cancer and P31 mesothelioma cells

**DOI:** 10.18632/oncotarget.21885

**Published:** 2017-10-16

**Authors:** Fintan Geoghegan, Robert J. Buckland, Eric T. Rogers, Karima Khalifa, Emma B. O’Connor, Mary F. Rooney, Parviz Behnam-Motlagh, Torbjörn K. Nilsson, Kjell Grankvist, Richard K. Porter

**Affiliations:** ^1^ School of Biochemistry and Immunology, Trinity Biomedical Science Institute (TBSI), Trinity College Dublin, Dublin 2, Ireland; ^2^ Dept of Medical Biosciences, Clinical Chemistry, Umeå University, Umeå, Sweden

**Keywords:** cisplatin resistance, bioenergetics, SIRT3, non-small cell lung cancer, mesothelioma

## Abstract

Acquired cisplatin resistance is a common feature of tumours following cancer treatment with cisplatin and also of non-small cell lung cancer (H1299) and mesothelioma (P31) cell lines exposed to cisplatin. To elucidate the cellular basis of acquired cisplatin resistance, a comprehensive bioenergetic analysis was undertaken. We demonstrate that cellular oxygen consumption was significantly decreased in cisplatin resistant cells and that the reduction was primarily due to reduced mitochondrial activity as a result of reduced mitochondrial abundance. The differential mitochondrial abundance was supported by data showing reduced sirtuin 1 (SIRT1), peroxisome-proliferator activator receptor-γ co-activator 1-alpha (PGC1α), sirtuin 3 (SIRT3) and mitochondrial transcription factor A (TFAM) protein expression in resistant cells. Consistent with these data we observed increased reactive oxygen species (ROS) production and increased hypoxia inducible factor 1-alpha (HIF1α) stabilization in cisplatin resistant cells when compared to cisplatin sensitive controls. We also observed an increase in AMP kinase subunit α2 (AMPKα2) transcripts and protein expression in resistant H1299 cells. mRNA expression was also reduced for cisplatin resistant H1299 cells in these genes, however the pattern was not consistent in resistant P31 cells. There was very little change in DNA methylation of these genes, suggesting that the cells are not stably reprogrammed epigenetically. Taken together, our data demonstrate reduced oxidative metabolism, reduced mitochondrial abundance, potential for increased glycolytic flux and increased ROS production in acquired cisplatin resistant cells. This suggests that the metabolic changes are a result of reduced SIRT3 expression and increased HIF-1α stabilization.

## INTRODUCTION

Altered metabolism is a hallmark of cancer [1] and while acquired genetic and molecular modifications have enabled cancers to survive the onslaught of various cancer therapies [2], understanding tumour cell metabolism is becoming increasingly more important. Changes to key processes like glucose, glutamine, nucleotide, fatty acid and phospholipid metabolism enable cancers to modify their growth demands and thrive in a hostile environment [3, 4]. Hence, metabolic features of cancers are currently being investigated not only to aid in the process of diagnosis but also with view to finding new therapeutic targets [5, 6].

Cisplatin is a potent chemotherapeutic drug, used in the treatment of a variety of cancers but its usefulness is limited by the acquisition of resistance. Cisplatin cytotoxicity has been generally attributed to its interaction with DNA to form intra-strand crosslinks [7–9]. Interestingly, some studies have indicated that mitochondrial DNA adducts may be more common than nuclear DNA adducts in model cell line systems as a result of cisplatin treatment [10, 11]. Resistant cancers have been shown to adapt to long-term cisplatin treatment by decreasing their mitochondrial number [12, 13], oxygen consumption and glucose uptake [14] and exploiting these metabolic differences may prove important in overcoming chemo-resistance. Also, acquired cisplatin resistant non-small cell lung cancer (H1299) and mesothelioma (P31) cell lines demonstrate reduced sensitivity to cisplatin-induced cell death when compared to their sensitive cell counterparts [15]. We decided to use these cells and apply comprehensive bioenergetics analyses to determine whether acquired cisplatin resistance was impacting mitochondrial function.

We therefore studied real-time *in situ* mitochondrial function, mitochondrial abundance and glycolytic flux. We compared mitochondrial biogenesis by analysing protein expression levels of cytosolic sirtuin 1 (SIRT1, NAD-dependent deacetylase), peroxisome-proliferator activator receptor-γ co-activator 1-alpha (PGC1α, central role in energy metabolism), transcription factor A, mitochondrial (TFAM, core mitochondrial protein) and sirtuin 3 (SIRT3, mitochondrial NAD-dependent deacetylase in the mitochondrial matrix associated with integrity/antioxidant responses). We investigated whether there was a correlation between acquired cisplatin resistance and HIF1α stabilization as had been identified by Ai *et al.* (2016) [16] in ovarian cells. We also looked at reactive oxygen species (ROS) production, as it can be augmented as a result of dysfunctional mitochondria through accumulations of mitochondrial mutations, impairment of oxidative phosphorylation and an imbalance in the expression of antioxidant enzymes [17]. In addition, we performed genome-wide transcriptome and epigenome (DNA methylation) analyses on the resistant vs. the parental cells, with the aim of getting a grasp of the mechanisms of the observed changes in the bioenergetics phenotypes.

## RESULTS

### Determination of the IC_50_ values for cisplatin in H1299, H1299r, P31 and P31r cells

**Figure 1:**
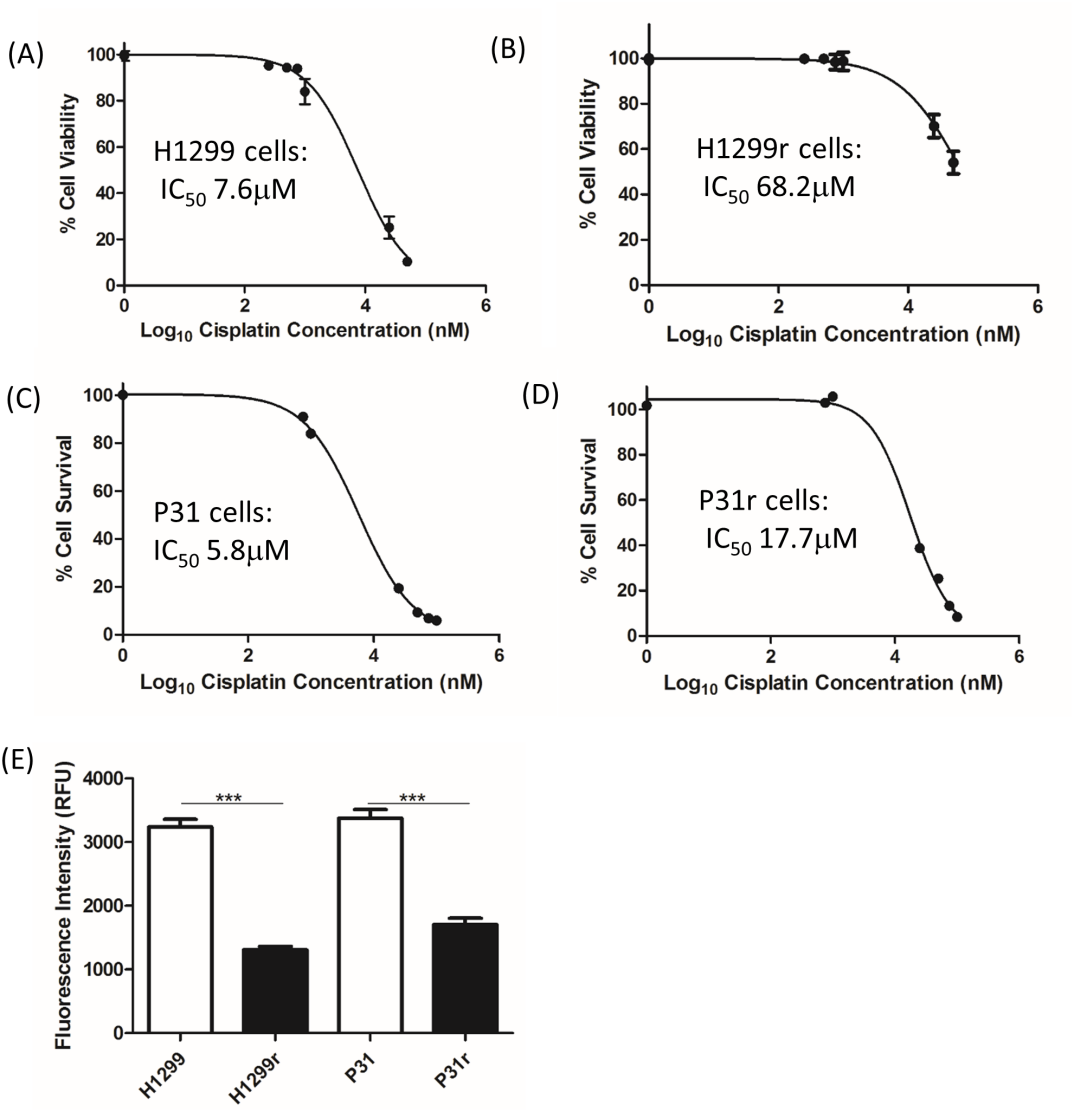
The effect of cisplatin on the viability of H1299, H1299r, P31 and P31r cells as determined by the Alamar Blue viability assay Cells were seeded in 96 well plates at the following densities **(A)** H1299, 2,000 cells/ well; **(B)** H1299r, 6,000/cell/well; **(C)** P31, 2,000 cells/ well; **(D)** P31r, 6,000 cells/ well. All cells were treated with vehicle (0.9% NaCl) or varying concentrations of cisplatin (50 nmol/L -100 μmol/L) for 72 h. Alamar blue was added and cells were incubated in the dark for 5 h. The fluorescence was read at an excitation wavelength of 544 nm and an emission wavelength of 590 nm using a micro plate reader. Data expressed as % cell viability of vehicle treated controls. IC_50_ values represent the concentration of drug required to reduce viability by 50 %. Data are expressed as mean ± SEM from three separate experiments, performed in triplicate. **(E)** The growth rate of the H1299, H1299r, P31 and P31r cells was assessed over 72 h by seeding cells at 2,000 cells/well in a 96 well plate. After the elapsed time 20 μL of Alamar blue was added to the wells and the fluorescence was then measured by a spectrophotometer. Data is expressed as fluorescence intensity. Data are expressed as mean ± SEM from three separate experiments. Statistical analysis was carried out using the student t-test. ^***^ = p<0.001.

In order to confirm the relative cisplatin sensitivities of the H1299 and P31 resistant and their parental counterparts, cells were treated with vehicle (0.9% NaCl) or varying concentrations of cisplatin (50 nmol/L -100 μmol/L) for 72 h and IC_50_ values were determined using the Alamar Blue viability assay. As seen in Figure 1, cisplatin decreased the viability of H1299, H1299r, P31 and P31r cells in a dose-dependent manner with the maximum cytotoxic effect being observed at approx. 100 μmol/L cisplatin. The IC_50_ value for cisplatin in the H1299 cells was 7.6 μmol/L (Figure 1A) and approx. 68.2 μmol/L (Figure 1B) for the H1299r cells. The IC_50_ value for cisplatin in the P31 cells was 5.8 μmol/L (Figure 1C) for the parental cells and 17.7 μmol/L (Figure 1D) for the resistant cells. Thus the H1299 resistant cells demonstrated a 10-fold greater resistance to cisplatin compared to the parental cells whereas the P31 resistant cells showed a 3-fold resistance to cisplatin compared to the sensitive cells. In addition, we observed that there was a significant (p<0.001) ∼2-fold greater proliferation rate in the parental cell lines when compared to the resistant cell lines (Figure 1E).

### Analysis of the whole cell metabolism of H1299, H1299r, P31 and P31r cell lines by the Seahorse extracellular flux analyser

Seeding optimisation first had to be performed as these cells had not been used on the Seahorse instrument before. The seeding densities were based upon the seeding density of another non-small cell lung cancer cell line, H460, which were seeded at 20,000 cells/well [18]. The H1299 and P31 cells were seeded at 10,000 cells, 20,000 and 30,000 cells/well all the while maintaining the resistant cells in cisplatin. As the trend between the sensitive and resistant cells was not drastically different between the different cell densities, it was decided to seed the cells at 20,000 cells/well.

**Figure 2:**
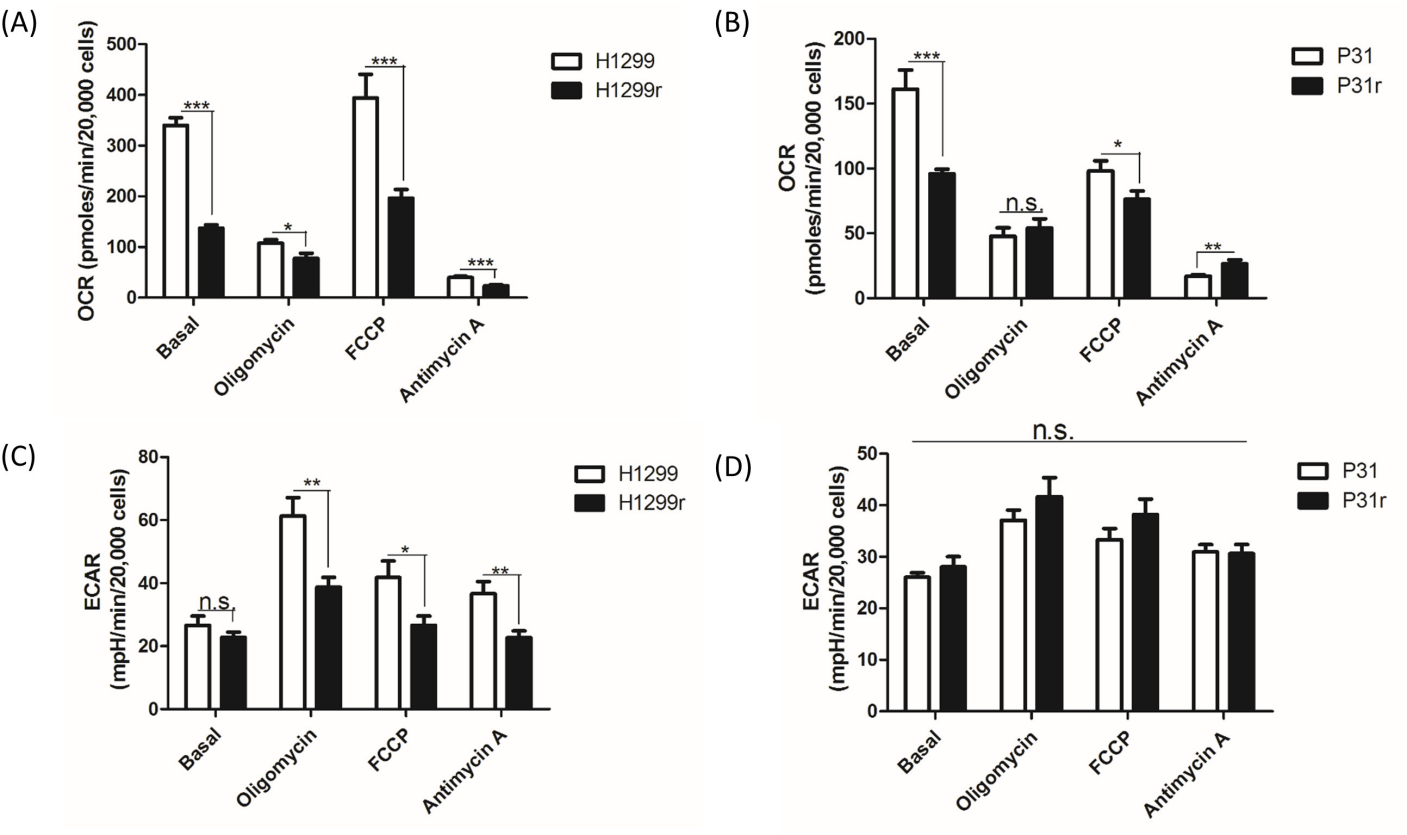
A comparative profile of whole cellular oxygen consumption rates and extracellular acidification rates for H1299, H1299r, P31 & P31r as determined by the Seahorse extracellular flux analyser Cells were seeded at 20,000 cells/well and an assay was performed using the Seahorse extracellular flux analyser where oligomycin, FCCP and antimycin A were sequentially added to the wells during the run. **(A)** Whole cell basal oxygen consumption rates (OCR) of H1299 and H1299r and **(B)** P31 and P31r cells were determined as well as cellular OCRs after the inhibitors/uncoupler were added to the wells. **(C)** Whole cell extracellular acidification rate (ECAR) of H1299 and H1299r and **(D)** P31 and P31r cells was determined as well as ECAR after the inhibitors/uncoupler were added to the wells. Data are expressed as mean ± SEM for three separate experiments. Statistical analysis was carried out using the student t-test. ^***^ = p< 0.001, ^**^ = p<0.01, ^*^ = p<0.05.

The bioenergetic status of the cells was then determined using cellular oxygen consumption rate (OCR) and extracellular acidification rate (ECAR) measurements. Basal oxygen consumption rates were OCRs without addition of any mitochondrial inhibitors or uncouplers. Oligomycin was added to inhibit mitochondrial ATP synthesis and under these conditions, one can determine the OCR response to the resulting lack of ATP turnover in mitochondria, and whether glycolysis (as measured by ECAR) increases in response to that inhibition. OCR when the mitochondrial electron transport chain is working maximally can sometimes be achieved by the addition of the mitochondrial uncoupler, FCCP (carbonyl cyanide-4-(trifluoromethoxy)phenylhydrazone). Finally, inhibition of mitochondrial function by addition of antimycin A allows one to dissect out the cellular oxygen consumption not due to oxidative phosphorylation and to determine how the glycolytic pathway flux responds to complete inhibition of mitochondrial function. Figure 2A gives a classic profile of cellular oxygen consumption for cisplatin H1299 and H1299r cells in the presence of inhibitors and uncoupler. OCRs in the presence of oligomycin were lower than basal rates; OCRs in the presence of uncoupler were greater than basal rates and a small but significant amount of cellular oxygen consumption was not due to oxidative phosphorylation. OCRs were significantly lower in H1299r cells compared to H1299 cells. Indeed there is a significant OCR decrease (c.50%) between the basal cellular (p<0.001), oligomycin treated (p<0.05), FCCP (maximal mitochondrial activity) (p<0.001) and antimycin A (oxygen consumption not due to mitochondria) (p<0.001) when comparing H1299 to H1299r cells (Figure 2A).

Figure 2B shows the profile of OCRs for cisplatin P31 and P31r cells in the presence of inhibitors and uncoupler. As expected, OCRs in the presence of oligomycin were lower than basal rates; however OCRs in the presence of uncoupler were lower than basal rates and, as in the H1299 cells, a small but significant amount of cellular oxygen consumption was not due to oxidative phosphorylation. Like in H1299 cells, both basal OCRs and OCRs in the presence of uncoupler FCCP were significantly lower in P31r cells compared to P31 cells, but no significant differences were observed in cellular oxygen consumption rates in the presence of oligomycin. Thus, in P31 cells, we see a significant decrease in the OCRs of the basal (p<0.001) and FCCP (p<0.05) treated cells but not in oligomycin treated cells (Figure 2B). Furthermore, it appears that non-mitochondrial OCRs or oxygen consumption not due to oxidative phosphorylation (plus antimycin A) is significantly greater (p<0.001) in P31r when compared to P31 cells.

It is also noteworthy that basal oxygen consumption is approximately 2-fold greater in H1299 cells, per unit number of cells, when compared to P31 cells (Figure 2A and 2B).

The ECAR is taken as an index of glycolytic flux, and as expected, oligomycin increased glycolytic flux in H1299r and H1299 cells compared to their respective basal ECAR rates. However, ECAR waned in H1299r and H1299 cells on addition of uncoupler or antimycin A (Figure 2C). However, ECAR was significantly lower in H1299r cells following oligomycin (p<0.01), FCCP (p<0.05) and antimycin A (p<0.01) treatment (Figure 2C) when compared to H1299 cells. By contrast, there was no significant difference between ECAR of P31 and P31r cells under basal conditions or following addition of inhibitors or uncoupler (Figure 2D). Interestingly, absolute basal ECARs were similar for both H1299r/H1299 and P31r/P31 cells (∼25 mpH/min/20,000 cells - Figure 2C and 2D).

In summary, these data demonstrate reduced cellular (and *in situ* mitochondrial) oxidative metabolism in acquired cisplatin resistant cells compared to their more cisplatin sensitive counterparts and reduced glycolytic flux after inhibition of ATP synthesis in H1299r cells compared to H1299 cells.

### Analysis of the mitochondrial content of H1299, H1299r, P31 & P31r cells

Having established that the basal and maximal *in situ* mitochondrial OCRs were lower in H1299r and P31r cells compared to the equivalent parental counterparts, we set out to determine whether the reduced *in situ* mitochondrial OCRs were due to reduced mitochondrial numbers in the cisplatin resistant cells. A variety of methods were used to determine mitochondrial content of the cells namely; (i) a citrate synthase assay, (ii) detection by immunoblotting of voltage dependent anion channel 1 (VDAC1 - a mitochondrial outer membrane protein) and (iii) immunoblotting of subunit 4 of cytochrome c oxidase, also a mitochondrial inner membrane protein.

**Figure 3:**
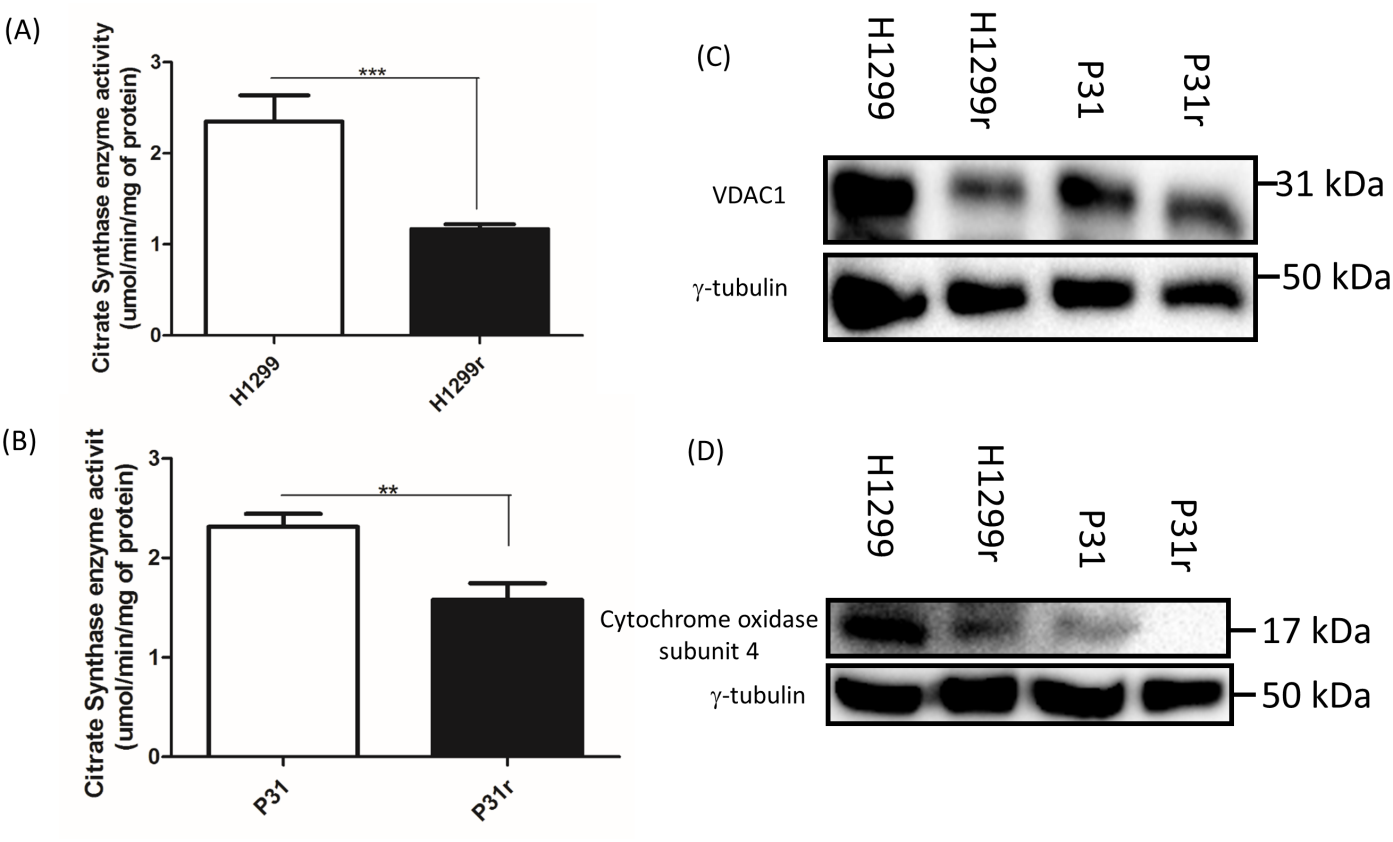
Comparative mitochondrial abundance as measured by citrate synthase activity, abundance of VDAC1 and cytochrome oxidase subunit 4 **(A)** Citrate synthase enzyme activity in the H1299 and H1299r cells. **(B)** Citrate synthase enzyme activity in the P31 and P31r cells. Data are expressed as mean ± SEM for three separate experiments. Statistical analysis was carried out using the student t-test. ^***^ = p< 0.001, ^**^ = p<0.01. For immunoblots, whole cell lysates were prepared from confluent cultures of H1299, H1299r, P31 and P31r cells. Proteins (90 μg) were resolved in 10% SDS-PAGE gels and transferred to a PVDF membrane. Blots were probed for either VDAC1, cytochrome c oxidase subunit 4 or the loading control γ-tubulin. **(C)** Representative blot of VDAC1 protein expression from three independent experiments. **(D)** Representative blot of cytochrome c oxidase subunit 4 protein expression from three independent experiments.

There was a significantly greater (∼2-fold) citrate synthase activity in the H1299 in comparison to H1299r cells (p<0.01) (Figure 3A). Similarly there was a significantly greater (∼1.5-fold) citrate synthase activity in the P31 cells in comparison to P31r cells (P<0.01) (Figure 3B). These quantitative results were mirrored by reduced protein levels of both VDAC1 and cytochrome c oxidase subunit 4 in both H1299r and P31r cells (Figure 3C and 3D). Taken together, the data clearly indicate reduced mitochondrial abundance in acquired cisplatin resistant cells compared to their more cisplatin sensitive parental counterparts.

### Qualitative profile of the expression of mitochondrial biogenesis proteins SIRT1, SIRT3, PGC-1α and TFAM in H1299, H1299r, P31 and P31r cells

**Figure 4:**
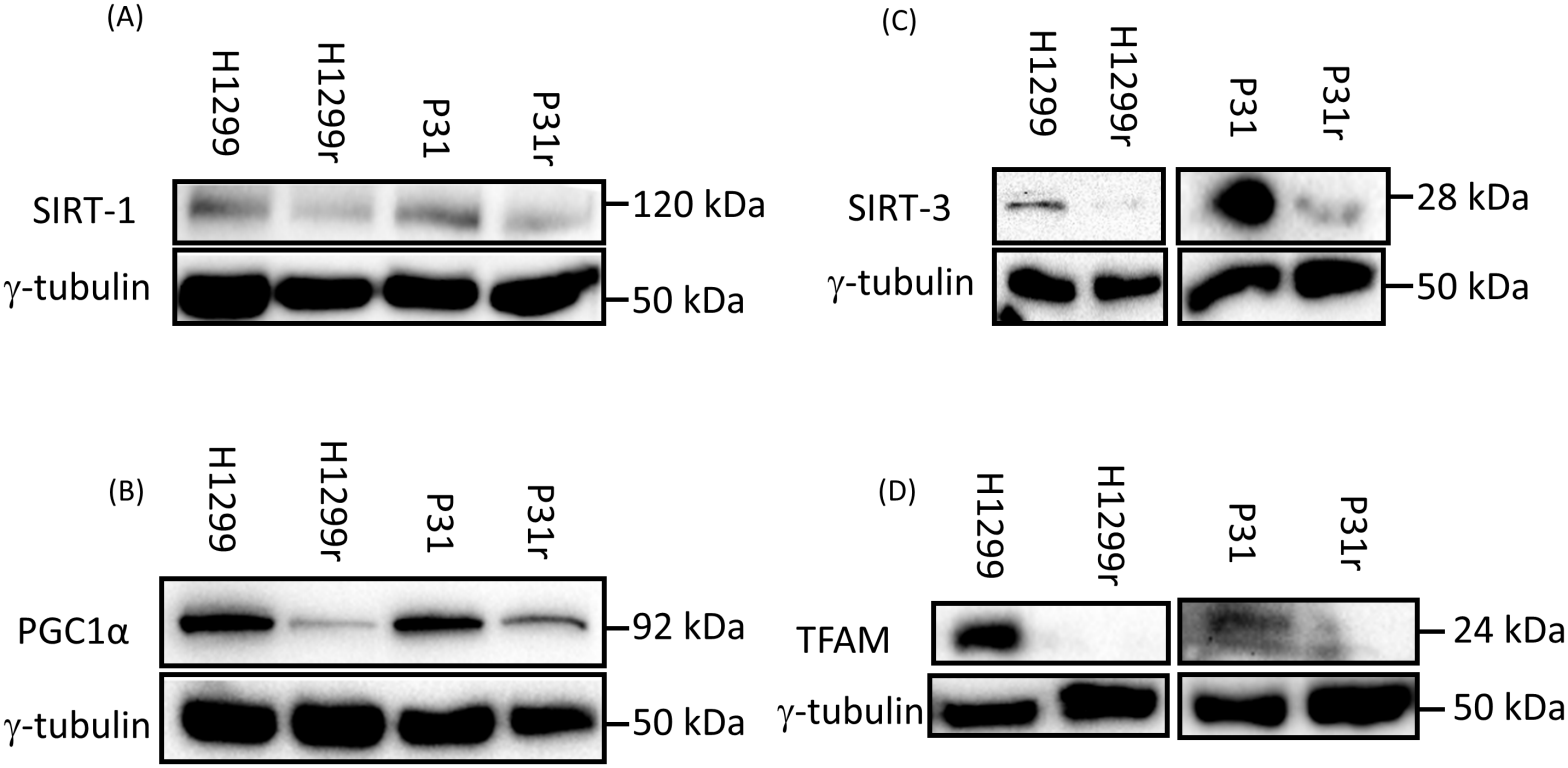
Expression of the mitochondrial biogenesis proteins: SIRT1, PGC1α, TFAM and SIRT3 in H1299, H1299r, P31 and P31r cell lysates as determined by immunoblot analysis Whole cell lysates were prepared from confluent cultures of H1299, H1299r, P31 and P31r cells. Proteins (90 μg) were resolved in 10% SDS-PAGE gels and transferred to a PVDF membrane. Blots were probed for SIRT1, PGC1α, TFAM and SIRT3 or the loading control γ-tubulin. Figure shows representative blots of **(A)** of SIRT1, **(B)** PGC1α, **(C)** SIRT3, **(D)** TFAM protein expression; each from three independent experiments.

Having demonstrated reduced mitochondrial activity and reduced mitochondrial abundance in cisplatin resistant cells compared to their cisplatin sensitive counterparts, we performed a qualitative profile, by immunoblotting, of proteins associated with mitochondrial biogenesis; cytosolic sirtuin 1 (SIRT1, NAD-dependent deacetylase) (Figure 4A), peroxisome-proliferator activator receptor-γ co-activator 1-alpha (PGC1α)(central role in energy metabolism) (Figure 4B), sirtuin 3 (SIRT-3, an NAD-dependent deacetylase in the mitochondrial matrix associated with integrity/antioxidant responses) (Figure 4C) and transcription factor A mitochondrial core mitochondrial protein(TFAM) (Figure 4D). These data provide supporting evidence for reduced mitochondrial abundance in cisplatin resistant cells compared to their cisplatin sensitive counterparts. This is further supported by the mRNA expression data (Table 1) for H1299 with large decreases seen for all four proteins in the resistant cells. However the picture is less clear in P31 cells, where mRNA expression decreased in SIRT1, but was practically unchanged for PGC1 and SIRT3, yet increased for TFAM.

**Table 1:** mRNA expression and DNA methylation of studied genes

Gene		Fold Change H1299r vs H1299		Fold Change P31r vs P31	
		mRNA Expression (Log2)	Methylation (Beta value)	mRNA Expression (Log2)	Methylation (Beta value)
CS	Citrate Synthase	-0,348	-0,045	0,573	-0,017
VDAC	Voltage-Dependent Anion Channel 1	0,226	-0,011	0,333	0,007
COX4	Cytochrome C Oxidase subunit IV isoform 1	-0,057	-0,017	0,261	-0,003
	Cytochrome C Oxidase subunit IV isoform 2 (lung)	0,129	0,021	0,206	-0,037
SIRT1	Sirtuin 1	-0,216	-0,077	-0,404	-0,011
SIRT3	Sirtuin 3	-0,098	-0,048	0,009	-0,072
PGC1α (PPARGC1A)	Peroxisome Proliferator-Activated Receptor Gamma, Coactivator 1 alpha	-0,371	0,004	0,056	-0,043
TFAM	Transcription Factor A, Mitochondria	-0,398	-0,026	0,556	-0,005
HIF1α	Hypoxia Inducible Factor 1, alpha subunit inhibitor	0,058	-0,008	-0,281	-0,007
AMPKα2 (PRKAA2)	Protein Kinase, AMP-activated, alpha 2 catalytic subunit	1,630	-0,091	0,077	0,200

Fold change (Log2) in mRNA expression between cells with acquired cisplatin resistance (r) and those without, measured using Affymetrix GeneChip^®^ HTA Arrays (GeneChip^®^ Human Transcriptome Array 2.0). Gene wide methylation Beta values (difference in methylation between cells with acquired cisplatin resistance (r) and those without measured using the Illumina Infinium 450k chip array.

The DNA methylation data shows only a slight decrease or no change in methylation status in all the four genes in both cell lines (Table 1) suggesting mechanisms other than DNA methylation are affecting mRNA and protein levels.

### Measurement of reactive oxygen species (ROS) in H1299 and H1299r as determined by flow cytometry

**Figure 5:**
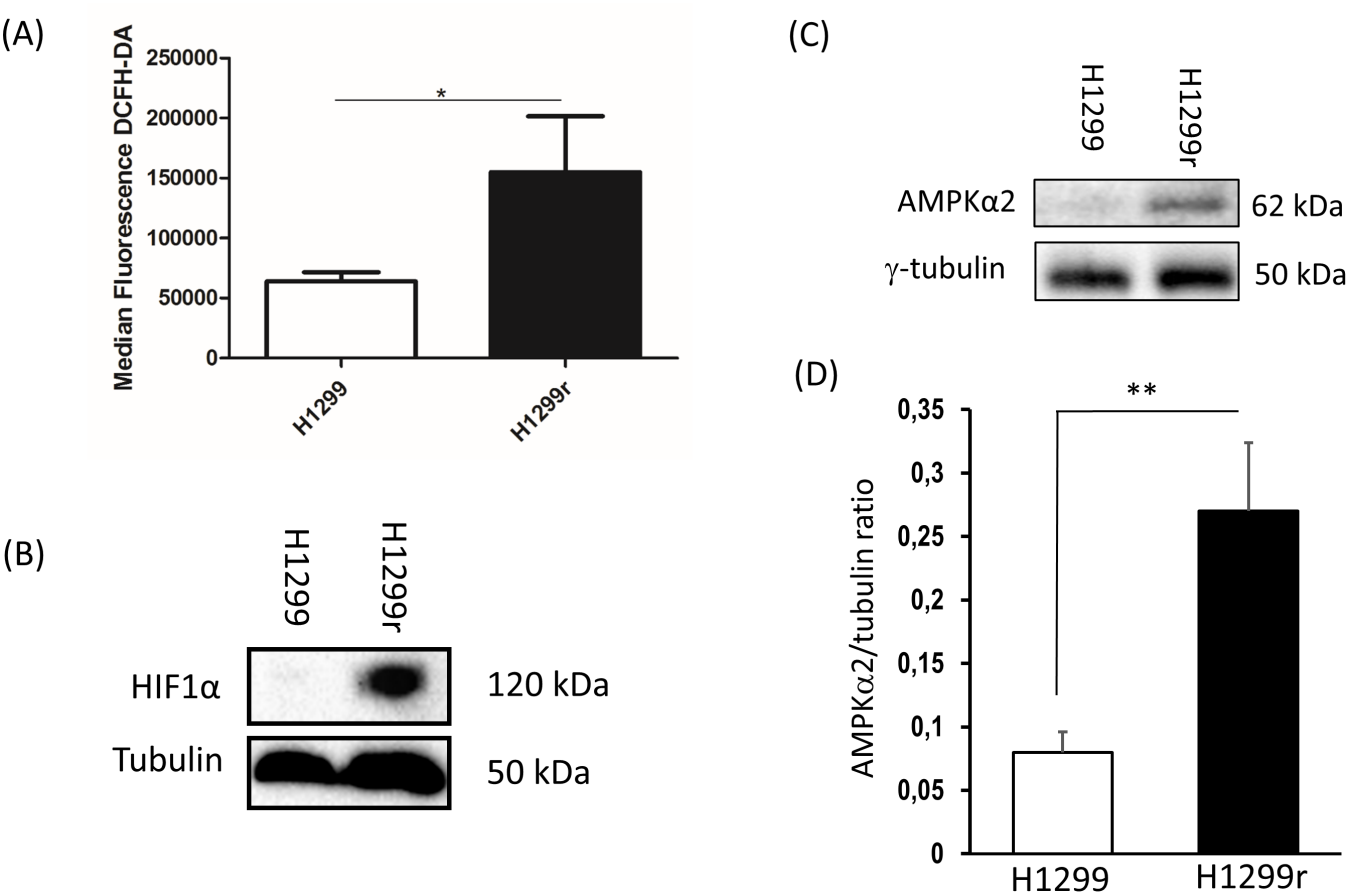
Reactive oxygen species production and expression of stabilized HIF1α and AMPK subunit α2 and in H1299 and H1299r cells **(A)** H1299, H1299r, P31 and P31r cell were seeded at a density of 250,000 cells/well and left for 24 h. The cells were then stained with 10 μmol/L dichloro-dihydro-fluorescein diacetate (DCFH-DA). Fluorescence was analysed for 10,000 events. Whole cell lysates were prepared from confluent cultures of H1299, H1299r cells. Proteins (90 μg) were resolved in 10% SDS-PAGE gels and transferred to a PVDF membrane. **(B)** Representative blot of HIF1α protein expression. **(C)** Representative blot of AMPK subunit α2 protein expression from four independent experiments. **(D)** Bar chart of AMPK subunit α2 mean for four separate measurements. Statistical analyses was carried out using the student t-test. ^**^ = p<0.01, ^*^ = p<0.05.

ROS production within the H1299 cells was measured using the intracellular fluorescent dye dichloro-dihydro-fluorescein diacetate (DCFH-DA). The H1299r cells exhibited a significantly greater (2-fold) degree of ROS production when compared to the H1299 cells (Figure 5A).

### Hypoxia inducible factor 1 alpha (HIF1α) stabilisation in H1299 and H1299r cells

Stabilized HIF1α transcription factor plays an essential role in increasing glycolytic flux and decreasing mitochondrial biogenesis in cancer cells [19]. Having observed the greatest decrease in mitochondrial activity and abundance in H1299r cells compared to H1299 cells, and having observed substantial increases in glycolytic flux (ECAR) after oligomycin addition in H1299r cells compared to H1299 cells we decided to compare HIF1α expression in these cells and not P31 cells. We observed a dramatic stabilized expression of HIF1α in H1299r cells compared to H1299 cells (Figure 5B). This large change in observed protein levels was not reflected in mRNA or DNA methylation where very little difference was seen between the resistant and wild type H1299 cells (Table 1).

### Comparative transcriptome and epigenome analysis

Results of a hypothesis driven comparative analysis of the mRNA expression and DNA methylation, are shown for selected genes of interest in Table 1. By far the largest difference in mRNA expression between resistant and sensitive cells was found in H1299 with the PRKAA2 gene (which codes for AMP Kinase subunit α2 (AMPKα2)). We were able to verify this differential expression at the protein level by immunoblot (representative blot, Figure 5C), demonstrating a significant 3-fold increase in AMPKα2 in H1299r cells compared to their parental controls (Figure 5D). The significance of these data will be covered in the discussion. The increase in mRNA expression in H1299r was accompanied by a 9% reduction in DNA methylation. It is interesting to note that there was very little change in mRNA expression (0.08 log fold) between the resistant and parental cells of P31 despite a larger and opposite change in methylation (+0.2 Beta). Overall, there was generally little change in the methylation of any of the metabolic control genes (see below) studied in either cell line (Supplementary Table 1). The mRNA expression data of the studied genes (Table 1) appears to highlight a difference between the two cell lines as most of the selected genes show a reduction with resistance in H1299 but an increase in P31. The exception is SIRT1 where mRNA expression decreased by 20-40% in both resistant cell lines. The difference in mRNA expression is further illustrated when a larger selection of metabolic control genes (Supplementary Table 1, based upon [20, 52]) was analysed, with the majority being non-concordant between the cell lines. We hypothesize that those genes that changed their expression in the same direction in both H1299 and P31 are more likely to be mechanistically involved in the induction of resistance than those genes that changed in opposite directions (Supplementary Table 1).

## DISCUSSION

We report here for the first time, real-time cellular metabolism comparing and contrasting oxidative and glycolytic fluxes in cisplatin-resistant and parental H1299 and P31 cells. No such data has been previously reported for these cells and acquired cisplatin resistance. Our data clearly demonstrate that basal cellular OCRs in parental H1299 and P31 cells are greater than oxygen consumption rates in resistant cells. Similar observations were made by Liang *et al.* [14] for human adenocarcinoma KB-3-1 and human hepatoma BEL7404 cells.

Acquired cisplatin resistance was approx. 10 fold in H1299 cells and 3 fold in P31 cells when compared to the parental cells. These data are consistent with data for cisplatin sensitivity reported for H1299 cells by Johansson *et al.* [15] and Huang *et al.* [21], for H1299r cells [15] and for P31 and P31r cells reported by Janson *et al.* [22] and Johansson *et al.* [15]. In addition, we observed that there was a significantly greater proliferation rate in the sensitive cell lines when compared to the resistant cell lines for both H1299 and P31 cells, an observation consistent with that measured previously for these cells by Tyler *et al.* [23].

Interestingly, the lowering of OCRs in resistant cells is not mirrored by an increase in extracellular acidification rate (ECAR), an index of glycolytic flux which one might expect if a lack of ATP synthesis by mitochondria is to be compensated by an increase in ATP production by glycolysis. Only when ATP synthesis is inhibited do we see a significant increase in glycolytic flux in resistant cells when compared to cisplatin sensitive cells, and even then only in H1299 cells. The data for H1299 cells suggests that acquired cisplatin resistance does not result in increased glycolytic flux, but if anything reduces the capacity for an increased glycolytic flux. This lack of glycolytic compensation in resistant cells, together with reduced oxidative phosphorylation, suggests that these (glucose and amino acid fuelled) cells are essentially ATP-deprived, as demonstrated by their lower growth rate.

Furthermore, the oxygen consumption not due to oxidative phosphorylation (non-mitochondrial oxygen consumption) is less in H1299r cells compared to H1299 cells, but greater in P31r cells compared to P31 cells. There are several possible reasons for this. The differential may reflect difference in activity of oxygen consuming enzymes such as monoamine oxidase, cytochrome P450 enzymes, haem oxygenase, xanthine oxidase and/or NADPH oxidase (NOX). H1299 cells are known to have an active NOX [24]. Furthermore, it is clear that H1299 cells are significantly (∼3 fold) more oxidative than P31 cells, yet glycolytic flux is similar in both cell types. These differences in oxidation between the cell lines could be indicative as to why H1299r cells have a higher (∼3 fold) resistance to cisplatin when compared to P31r cells.

Next we determined whether the decreased oxygen consumption in acquired cisplatin resistant cells was due to reduced *in situ* mitochondrial activity. There was approximately a 3-fold reduction in mitochondrial activity in H1299r cells compared to H1299 cells under basal and maximal activity conditions. Similarly, there is a reduction in *in situ* mitochondrial activity in P31r cells compared to P31, but to a lesser extent than in H1299 cells. The permeability of mitochondria was equivalent in cisplatin resistant and parental cells for both cell types, implying that differential mitochondrial activity between cisplatin resistant and parental cells is not due to differences in mitochondrial inner membrane leakiness. One interpretation of the data would be that the mitochondria in H1299r cells have a greater capacity to do more work than their cisplatin sensitive counterparts, so some of the reduced activity in cisplatin-resistant H1299 cells is due to reduced ATP turnover, whereas the mitochondria in P31 cells seem to be at maximal capacity under basal conditions. Having said that, there also remains the possibility that FCCP may have secondary effects on bioenergetics. For instance, the lower than expected oxygen consumption in the presence of oligomycin and FCCP may underestimate maximal oxidative capacity in the time frame measured.

Apart from reduced ATP turnover another possibility for the reduced *in situ* mitochondrial activity in acquired cisplatin-resistant cells compared to parental cells would be reduced mitochondrial abundance. Indeed we found reduced mitochondrial abundance in cisplatin resistant H1299r and P31r cells compared to their parental counterparts. Previous researchers have described reduced mitochondrial numbers in acquired cisplatin resistant human adenocarcinoma KB-3-1 and human hepatoma BEL7404 cells [14], and human gastric cancer cells [25], while Qian *et al.* [12] observed that cisplatin sensitivity in intestinal epithelial cells (IEC-6) directly correlated with mitochondrial abundance. The decrease in mitochondrial number may be one of the mechanisms by which these cancer cells have acquired resistance to cisplatin and clearly other factors such as differential multidrug resistance expression levels have been demonstrated for these cells [15]. However, reduction in mitochondrial number has been associated with tumour progression and prognosis in patients with hepatocellular carcinoma and breast cancer [13, 26]. A decrease in mitochondrial number was significantly associated with the development of metastasis in Ewing’s sarcoma [27]. The more aggressive cancers possess less mitochondria as they use glycolysis as their primary source of energy [28]. Another mechanism that has been suggested as a way of coping with low ATP levels is that cancer cells may internalize extracellular ATP [29].

The process of mitochondrial biogenesis involves the coordinated expression of nuclear and mitochondrial encoded gene products and replication of the mitochondrial genome [30, 31]. Crucial nuclear encoded proteins such as TFAM, are translated in the cytosol of the cells and contain a mitochondrial targeting sequence which enables their regulated import into the mitochondrial network where they are sorted according to their function [32]. PGC1α, a multifunctional transcriptional co-activator, regulates the expression of TFAM, a gene that mirrors the changing levels of mitochondrial DNA (mtDNA) and plays a crucial role in mtDNA maintenance [33]. PGC1α is known as the main mediator of transcriptional responses to nutrient stress, promoting mitochondrial biogenesis, cellular metabolism, and antioxidant responses through coordinated activation of the transcription factors NRF2, ERRα and PPARγ [30, 34]. PGC1α is controlled at the post-translational level by both phosphorylation by AMPK and deacetylation by a NAD+ dependent SIRT1. Interestingly, in this context, we do see an increase in AMPK α subunit 2 in resistant H1299 cells, but the relevance of this observation is not clear. Deacetylation by SIRT1 supports mitochondrial biogenesis and maintains the sensitivity of PGC1α to both the energy and redox balance within the cell [35]. Dual activation of both PGC1α and ERRα, promotes the expression of the downstream mitochondrial SIRT3 that ensures proper scavenging of ROS by activation of mitochondrial superoxide dismutase, as well as other mitochondrial sirtuin targets [36]. In our study, the expression levels of SIRT1, PGC1α, SIRT3 and TFAM proteins involved in mitochondrial biogenesis were measured. We demonstrated that protein expression of all four proteins were reduced in the cisplatin resistant cells of the H1299 and P31 cells when compared to their parental counterparts, a pattern that was repeated in the mRNA expression data for H1299 but not in P31 cells where TFAM increased. SIRT1 expression and activity has been positively associated with drug resistance [37]. Contrary to our observations, Liang *et al.* [14] have demonstrated increased SIRT1 expression in acquired cisplatin resistant human adenocarcinoma KB-3-1 cells and human hepatoma BEL7404 cells. Based on the aforementioned role of SIRT1 in mitochondrial biogenesis, our results are consistent with the logic that a reduced SIRT1 activity, would result in less deacetylation of PGC1α and thus reduced mitochondrial biogenesis, which is what we observed. The data for PGC1α are in agreement with others who have shown reduced PGC1α expression in breast tumour tissue [38], colon cancer [39] and in human hepatocellular carcinoma cells [40]. The data for TFAM are also consistent with data from other researchers who show that TFAM and PGC1α levels are reduced in the acquired cisplatin resistant ovarian clear-cell carcinoma subline SKOV-3-R when compared to parental controls [41]. The large difference between the two cell lines in mRNA expression of TFAM (beta values of -0.4 in H1299 and +0.56 in P31 when comparing resistant to sensitive) suggest that a different translation mechanism is responsible for the reduction in this protein’s expression seen in both resistant cell lines.

The DNA methylation data suggests that gene-level methylation is not a major factor in the expression of these 4 genes as there is very little difference between the acquired cisplatin resistant and parental cells. This also suggests that the resistant phenotype could be a reversible trait.

Selecting the more oxidative H1299 cells for further studies, we were also able to demonstrate that there was greater ROS production in H1299r cells compared to H1299 cells. These data are consistent with the fact that reduction in SIRT3 is associated with increased reactive oxygen species production [42]. Our data are also consistent with those of Qian *et al.* [12] who demonstrated mitochondrial dysfunction in intestinal epithelial cells following long-term cisplatin treatment, and consistent with an imbalance in the expression of antioxidant enzymes as observed by Wang *et al.* [17]. Furthermore, SIRT3 also suppresses HIF1-α and its targeted genes as has been demonstrated in human colon carcinoma, osteosarcoma cells [42] and breast cancer cells [43]. As we had seen reduced oxidative metabolism, reduced mitochondrial abundance, potential for increased glycolytic flux, increased ROS production and reduced SIRT3 expression in acquired cisplatin resistant H1299 cells when compared to H1299 cells, we predicted that we would see increased stabilized HIF1-α in H1299r cells which was confirmed in H1299r cells by immunoblot. Increased AMPKα2 mRNA expression was also observed in H1299r cells (Table 1), where it showed the largest change of any studied gene. As already mentioned, the relevance of the α2-subunit in the context of acquired cisplatin resistance is not clear. Despite the huge rise in mRNA expression and increased protein expression of AMPKα2 seen in H1299r, there is only a slight change in methylation. Further studies are required to determine why that particular subunit was preferentially expressed in H1299r cells over H1299 cells. The lack of concordant changes in mRNA expression found between the two cell lines suggests different mechanisms of acquiring resistance to cisplatin between the two cell lines which could also account for the fact that H1299r is approximately 3 times more resistant to cisplatin than P31r.

In summary, we have demonstrated reduced oxidative metabolism, reduced mitochondrial abundance, potential for increased glycolytic flux and increased ROS production in acquired cisplatin resistant cells. The aforementioned metabolic changes most likely manifest as a result of reduced SIRT3 expression and increased HIF1-α stabilization. These differential metabolic indices are also partially evident in P31 mesothelioma cells. As reduced mitochondrial function/abundance seems to be a hallmark and probably a cause of acquired cisplatin resistance, we are the first to demonstrate this phenomenon in non-small lung cancer H1299 cells and P31 mesothelioma cells.

## MATERIALS AND METHODS

### Materials

Fetal bovine serum (FBS), trypsin-EDTA 0.25% solution, Dulbecco’s phosphate buffered saline (PBS), Dulbecco’s Modified Eagle Medium (DMEM) F12 + Nut mix, and Alamar blue assay kit were from Thermo Fisher Scientific, Inc. Antimycin A, rotenone, oligomycin, carbonyl cyanide-*4*-(trifluoromethoxy)phenylhydrazone (FCCP), sodium dodecyl sulphate (SDS), N,N,N’,N’-tetramethylethylenediamine (TEMED), immobilon transfer membrane, dimethylsulfoxide (DMSO), 5,5’-dithiobis(2-nitrobenzoic acid), 2’,7’ –dichlorofluorescin diacetate (DCFDA), cisplatin, *N*-acetylcysteine and hydrogen peroxide were obtained from Sigma Aldrich (Thermo Fisher Scientific, Inc., Waltham, MA, USA). Immobilon Western chemiluminescence HRP substrate was from Millipore. Penicillin streptomycin was from Invitrogen. Antibodies used for immunoblotting were AMPK α2 subunit (Cell Signalling Inc., catalogue number 2757S), sirtuin 1 (Cell Signalling Inc., catalogue no. 9475P), sirtuin 3 (Cell Signalling Inc., catalogue no. 5490), TFAM (Cell Signalling Inc., catalogue no. 7495S), γ-tubulin (Novus Biologicals, catalogue no. TU-32), PGC1α (Novus Biologicals, catalogue no. NBP1-04676), hypoxia inducible factor 1α (HIF1α) (Novus Biologicals catalogue no. NB100-449), Voltage dependent anion channel 1 (VDAC1) (Abcam catalogue no. ab15895), cytochrome oxidase subunit 4 (custom made for RKP by Eurogentec, Belgium), horseradish peroxidase (HRP)-conjugated anti-mouse (Promega catalogue no. W4021), anti-rabbit (Promega catalogue no. W4011), Fc block (BD Pharmingen catalogue no. 564220). Immobilon Western Chemiluminescence HRP substrate (EMD Millipore) was used for protein detection.

### Culturing H1299, H1299r, P31 and P31r cells

The non-small cell lung cancer parental cell line H1299, the induced acquired cisplatin resistant cell line H1299r, the mesothelioma parental cell line P31 and the cisplatin resistant mesothelioma cell line P31r were seeded and cultured as previously described [8]. H1299 and P31 cells were maintained in modified Eagle’s medium (MEM) supplemented with 2 % (w/v) penicillin streptomycin and 10% FBS. H1299r cells were maintained in MEM medium supplemented with 2 mg/L of cisplatin, 2 % (w/v) penicillin streptomycin and 10% FBS. P31r cells were maintained in MEM supplemented with 1.2 mg/L of cisplatin, 2 % (w/v) penicillin streptomycin and 10% (v/v) FBS. Concentrations of cisplatin used for the IC_50_ analysis ranged from 50 nmol/L -100 μmol/L. H1299 cells were originally established from a lymph node metastasis of a non-small cell lung cancer (NSCLC) [44] and the P31 cell line was originally established from a male patient with a pleural malignant mesothelioma tumour [45].

### Alamar blue viability assay

The cell proliferation assay was performed using the AlamarBlue^®^ assay (Medical Supply Company, Ltd., Dublin, Ireland) according to the manufacturer’s protocol. The cells were plated at 4,000 cells/well in triplicate in a 96-well, cultured overnight at 37°C in 5% CO_2_. Alamar blue (10% v/v, 20 μL) was added to the wells for 3-5 h. Fluorescence was analysed using Spectra Gemini microplate reader (Molecular Devices, LLC, Sunnyvale, CA, USA) at a wavelength of 544 nm with a reference wavelength of 590 nm. Results were presented as the percentage of viability relative to the vehicle control (100%). Dose response curves and IC_50_ determination were analyzed using Prism GraphPad version 5 software (GraphPad Software, Inc., La Jolla, CA, USA).

### Extracellular acidity rate and oxygen consumption rate

Cells were seeded at 20,000 cells/well and were cultured overnight in Seahorse extracellular flux analyser 24-well plates (Agilent). The plate was placed in the Seahorse extracellular flux analyser (Agilent Technologies, Santa Clara, USA) and extracellular acidity rate and oxygen consumption rate were measured simultaneously. It should be noted that ECAR is not necessarily a true reflection of absolute glycolytic rate as extracellular acidification would include acidification due to carbon dioxide production [46].

### Citrate synthase assay

H1299, H1299r, P31 and P31r cells were plated at 300,000 cells in T25 culture flasks, lysed with Tris-Triton X-100 (0.2% (v/v)) and the protein content was assessed using the BCA assay (below). 40 μg of protein was added into the cuvette with 500 μL of Tris (200 mmol/L, pH 8.0) with Triton X-100 (0.2% (v/v)), 100 μL of 5,5-dithio-bis-(2-nitrobenzoic acid)(DTNB) and 30 μL of acetyl coenzyme A (10 mmol/L). The coupled reaction was started by adding 50 μL of 10 mmol/L oxaloacetic acid and measuring the rate of increase in 5-thionitrobenzene absorbance at 412 nm for 3 min in a Spectra max 340PC spectrophotometer (Molecular Devices).

### Western blot analysis

Cells were lysed in Laemelli buffer [2% (w/v) SDS, 10% (v/v) glycerol, 60 mmol/L Tris-HCL pH 6.8]. Cell lysate protein concentrations were determined by using a bicinchoninic acid (BCA) assay (Pierce Biotechnology, Inc., Illinois, USA) and a Spectra max 340PC spectrophotometer (Molecular Devices). Samples protein were separated by 10% and 5–14% Mini-PROTEAN TGX gels (Bio-Rad, Ca, USA). The separated protein fractions in the gels were then transferred to PVDF membranes (Midi format 0.2 μm, Bio-Rad, USA) using Trans-Blot Turbo Transfer System (Bio-Rad, USA). The membranes were blocked in Marvel (low fat milk powder, Premier Foods, UK) for 1 h at 21°C, washed with PBS Tween (PBST) and subsequently incubated with antibody for AMPK subunit α2 (1:1000), HIF-1α (1:500), SIRT1 (1:500), PGC1α (1:1000), TFAM (1:500), SIRT3 (1:1000), VDAC1 (1:1000), cytochrome oxidase subunit 4 (1:500), or the loading control γ-tubulin (1:2000), diluted in 1% PBST at 4°C overnight. The membranes were washed with PBST before they were incubated diluted 1:10,000 in 1% PBST for 1 h at 21°C. Secondary antibodies used were either horseradish peroxidase (HRP)-conjugated anti-rabbit (1:2500) or HRP-conjugated anti-mouse (1:2500) secondary antibody. After washing with PBST the proteins were visualized using a Bio-Rad Chemi-Doc MP, Imaging System. Chemiluminescence HRP substrate (EMD Millipore) was used for protein detection. Western blots were normalised to γ-tubulin and densitometry analysis of bands was performed using ImageJ software (version 1.47v) (National Institute of Health, USA).

### Measurement of reactive oxygen species (ROS) by flow cytometry

H1299, H1299r, P31 and P31r cell were seeded at a density of 250,000 cells/well and cultured for 24 h. The cells were then stained with 10 μM dichloro-dihydro-fluorescein diacetate (DCFH-DA). The cell permeant DCFDA (2’,7’ –dichlorofluorescin diacetate) was used to measure hydroxyl, peroxyl and other reactive oxygen species (ROS) activity within the cell. DCFDA is deacetylated by cellular esterases to a non-fluorescent compound, which is oxidized by ROS into 2’, 7’ –dichlorofluorescein (DCF). DCF is highly fluorescent and is detected by fluorescence spectroscopy with maximum excitation and emission spectra of 495 nm and 529 nm, respectively. Fluorescence was analysed by flow cytometry (CYAN ADP Analyser, Beckman Coulter).

### Microarray transcriptome analysis

RNA concentration was measured with an ND-1000 spectrophotometer (NanoDrop Technologies, Wilmington, DE) and RNA quality was evaluated using the Agilent 2100 Bioanalyzer system (Agilent Technologies Inc., Palo Alto, CA). Total RNA (250 ng) from each sample was used to generate amplified and biotinylated sense-strand cDNA from the entire expressed genome according to the GeneChip^®^ WT PLUS Reagent Kit User Manual ( P/N 703174 Rev 1 Affymetrix Inc., Santa Clara, CA). GeneChip^®^ HTA Arrays (GeneChip^®^ Human Transcriptome Array 2.0) were hybridized for 16 h at 45°C, with 60 rpm rotation. According to the GeneChip^®^ Expression Wash, Stain and Scan Manual (PN 702731 Rev 3, Affymetrix Inc., Santa Clara, CA) the arrays were then washed and stained using the Fluidics Station 450 and finally scanned using the GeneChip^®^ Scanner 3000 7G.

The raw data was normalized in the free software Expression Console provided by Affymetrix (http://www.affymetrix.com) using the robust multi-array average (RMA) method [47, 48].

### Genome-wide DNA methylation data analysis

This was measured using the Illumina Infinium 450k chip array (San Diego, CA, USA). Raw data was provided from the SNP & SEQ technology Platform (SciLifeLabs) at Uppsala University, in the form of IDAT-files. Colour balance adjustment and background correction was performed using the methylumi package available from the Bioconductor project (www.bioconductor.org), in the freely available statistical computing language R (http://www.r-project.org).

Quantile normalization of the beta-values was carried out in the R-package wateRmelon [49] in order to reduce for the sample-to-sample variation. This was followed by beta mixture quantile dilation (BMIQ), a method for eliminating the probe type bias in the Illumina Infinium technology [50]. Filtering was done in the same package, where sites with bead count < 3 in more than 5% of the samples were removed, together with sites where more than 1% of samples had a detection p-value > 0.05. Further, probe filtering was performed according to the Supplementary Table 1 from Nordlund *et al.* [51], where probes in proximity to SNPs or at SNP-sites were removed. Probes on X and Y chromosomes were retained. Annotation was performed with the R-package COHCAP. Finally, the beta-values for the remaining 446 412 probes were presented and an average beta-value for each gene was calculated.

### Statistical analysis

The statistical analysis of experimental data was performed using the computer program GraphPad Prism 5 (GraphPad Software, La Jolla, CA, USA). Unless otherwise stated, data are expressed as the mean ± standard error of the mean (SEM) from three separate experiments performed in triplicate. Statistical analysis was performed using an unpaired Student’s t-test. P<0.05 was considered to indicate a statistically significant difference (^*^≤0.05; ^**^≤0.01; ^***^≤0.001)

## SUPPLEMENTARY MATERIAL TABLE





## Abbreviations

OCR: oxygen consumption rate
ECAR: extracellular acidification rate
