# Supplementary material for: Bioenergetics of acquired cisplatin resistant H1299 non-small cell lung cancer and P31 mesothelioma cells
Source: Oncotarget. 2017 Oct 16;8(55):94711–25. doi: 10.18632/oncotarget.21885 (PMC5706906; doi:10.18632/oncotarget.21885)
Supplement: Supplementary file 1 [file oncotarget-08-94711-s001.pdf]

Bioenergetics of acquired cisplatin resistant H1299 non-small cell lung cancer and P31 mesothelioma cells

SUPPLEMENTARY MATERIALS

Supplementary Table 1: mRNA expression and DNA methylation of metabolic control genes

Gene list based upon Kroemer *at al*. reviews [20, 52]. Fold change (Log2) in mRNA expression between cells with acquired cisplatin resistance (r) and those without, measured using Affymetrix GeneChip® HTA Arrays (GeneChip® Human Transcriptome Array 2.0). Gene wide methylation Beta values (difference in methylation between cells with acquired cisplatin resistance (r) and those without measured using the Illumina Infinium 450k chip array.

See Supplementary File 1
